# Identification of novel sources of partial and incomplete hypersensitive resistance to rust and associated genomic regions in common bean

**DOI:** 10.1186/s12870-023-04619-8

**Published:** 2023-12-01

**Authors:** Susana Trindade Leitão, Diego Rubiales, Maria Carlota Vaz Patto

**Affiliations:** 1 Instituto de Tecnologia Química e Biológica António Xavier, Universidade Nova de Lisboa (ITQB NOVA), Oeiras, 2780-157 Portugal; 2 Institute for Sustainable Agriculture, CSIC, 14004 Córdoba, Spain

**Keywords:** *Phaseolus vulgaris*, *Uromyces appendiculatus*, Partial resistance, Hypersensitive resistance, Association mapping

## Abstract

Common bean (*Phaseolus vulgaris*) is one of the legume crops most consumed worldwide and bean rust is one of the most severe foliar biotrophic fungal diseases impacting its production. In this work, we searched for new sources of rust resistance (*Uromyces appendiculatus*) in a representative collection of the Portuguese germplasm, known to have accessions with an admixed genetic background between Mesoamerican and Andean gene pools. We identified six accessions with incomplete hypersensitive resistance and 20 partially resistant accessions of Andean, Mesoamerican, and admixed origin. We detected 11 disease severity-associated single-nucleotide polymorphisms (SNPs) using a genome-wide association approach. Six of the associations were related to partial (incomplete non-hypersensitive) resistance and five to incomplete hypersensitive resistance, and the proportion of variance explained by each association varied from 4.7 to 25.2%. Bean rust severity values ranged from 0.2 to 49.1% and all the infection types were identified, reflecting the diversity of resistance mechanisms deployed by the Portuguese germplasm.

The associations with *U. appendiculatus* partial resistance were located in chromosome Pv08, and with incomplete hypersensitive resistance in chromosomes Pv06, Pv07, and Pv08, suggesting an oligogenic inheritance of both types of resistance. A resolution to the gene level was achieved for eight of the associations. The candidate genes proposed included several resistance-associated enzymes, namely β-amylase 7, acyl-CoA thioesterase, protein kinase, and aspartyl protease. Both SNPs and candidate genes here identified constitute promising genomics targets to develop functional molecular tools to support bean rust resistance precision breeding.

**Supplementary Information:**

The online version contains supplementary material available at 10.1186/s12870-023-04619-8.

## Background

Common bean (*Phaseolus vulgaris* L.) is one of the most important food crops worldwide, with more than 27 million tons produced in 2020 (FAOSTAT). In Portugal, it is the most consumed grain legume, despite the national production covering only around 10% of the country’s demand (“Estatísticas Agrícolas 2020”, www.ine.pt). Different reasons are responsible for the reduced common bean cultivation in Portugal and Europe, e.g., low productivity of existing varieties and little investment in breeding to overcome the unstable yields caused, among other factors, by susceptibility to pests and diseases [1, 2]. Nevertheless, Portugal has diverse underexploited common bean genetic resources with the potential to overcome the challenge of stabilizing/increasing the sustainability of yields under a changing climate and decreasing the dependency on imports [1, 3–5]. Common bean has been cultivated in Portugal since the sixteenth century, in low-input farming systems, mainly for farmers’ self-consumption or to sell in local markets [6, 7]. The analysis of the Portuguese germplasm’s genetic structure and phaseolin type diversity [3] revealed its proximity to the Andean gene pool and that one-third had an admixture origin (between the Andean and Mesoamerican gene pools), bearing unique genetic combinations with potential for common bean breeding. For example, complete and incomplete sources of resistance to fusarium wilt were already identified among those accessions of admixed origin [4].

Bean rust is an important disease, caused by the airborne, obligate biotrophic fungus *Uromyces appendiculatus* (Pers.) Unger, responsible for yield losses of up to 69% depending on the cultivar, crop age at the time of infection, and environmental conditions [8]. As obligate biotrophs, rust fungi can only feed, grow, and reproduce on their living hosts. The main host of *U. appendiculatus* is common bean, but it also infects other *Phaseolus* spp. such as *P. coccineus* (runner bean) or *P. lunatus* (lima bean), as well as *Vigna* spp., such as *V. unguiculata* (cowpea) [9]. Infection begins with stomata penetration, followed by contact with host mesophyll cells and haustorium formation within the host cell cavity [10]. Disease symptoms initially appear as small yellow spots on the adaxial and/or abaxial surfaces of the leaves. A few days later those spots enlarge and give rise to rusty-brown pustules (uredia). Sporulation begins and urediniospores may attain a diameter of 1–2 mm about 8–12 days after inoculation [11]. Infection occurs mainly in leaves, but symptoms may appear also in stems, branches, and pods. Hypersensitive resistance (HR) based on host cell death has frequently been described in many pathosystems, being typically monogenic and considered qualitative in expression [12]. Monogenic resistance is typically based on the hypersensitive response, which can be early-acting and complete, or late-acting and incomplete, allowing some rust development, and therefore, providing only incomplete levels of resistance, with reduced disease severity (DS) but with rust pustules associated with some levels of necrosis (reduced infection type, IT), any case, being race specific. This should not be confounded with incomplete resistance not based on hypersensitivity, also called partial resistance [13] which is typically oligogenic and race-non-specific. In partial resistance, plants are compatible with the pathogen showing a high IT score, despite a reduced sporulation of the fungus (reduced DS) [14]. Both types of resistance can be identified in any crop, but monogenic, complete resistance is usually preferred by breeders as it is easier to identify and handle in a breeding program [15].

Monogenic resistance to rust has been widely deployed in common bean breeding, with 14 dominant major resistance genes identified, 10 of which have been characterized, and mapped in the *P. vulgaris* genome (Miklas 2002): *Ur-3* (in chromosome Pv11), *Ur-5* (in Pv04), *Ur-7* (in Pv11), *Ur-11* (in Pv11) and *Ur-14* (in Pv04) belonging to the Mesoamerican gene pool, and *Ur-4* (in Pv06), *Ur-6* (in Pv11), *Ur-9* (in Pv01), *Ur-12* (in Pv07) and *Ur-13* (in Pv08) belonging to the Andean gene pool [16, 17]. Those rust resistance genes and closely linked markers were successfully used in marker-assisted backcrossing for the development of resistant varieties [11, 16, 18, 19]. However, monogenic resistance may be rapidly overcome by new races of the pathogen and as a result, more than 90 races of *U. appendiculatus*, have been identified [20, 21]. Indeed, the rust fungus is among the highest-risk pathogens for breaking down the effectiveness of resistance genes due to its fast and effective air diffusion and the coexistence of sexual and asexual reproduction cycles [22]. As already pointed out, the development of resistant common bean cultivars based only on single resistance genes might not be appropriate to attain durable resistance, as the rust pathogen easily evolves and breaks down the resistance. For example, resistance genes *Ur-1*, *Ur-2*, and *Ur-8,* identified 40 years ago, have been overcome by the majority of *U. appendiculatus* races and are no longer used by breeders as sources of resistance [16].

The combination of different resistance genetic basis remains the most cost-effective control measure for creating broad resistance to the multiple races of rust pathogens [23]. For that, different approaches may be followed: several major resistance genes may be pyramided into a single cultivar to hinder the pathogen from undergoing a sequence of mutations corresponding to each resistance gene. Alternatively, one can rotate major gene resistance through time and space or grow cultivars with different resistance genes [22]. However, despite this difficulty, it is highly desirable to identify and characterize incomplete resistance for its potential durability. The combination of minor and major genes and genes acting at different levels of resistance mechanism is also promising because it relies on multi-resistant alleles to confer a more durable resistance [24, 25]. Availability of molecular markers for the various major and minor genes is therefore critical for achieving affordable selection and combination of resistance loci under precision breeding [26, 27].

Since the release of a reference genome for *Phaseolus vulgaris* [28] and with an increasing diversity of SNP arrays available, genome-wide association studies (GWAS) have been employed to detect genetic variations related to disease resistance in common bean [29–32]. GWAS was previously successfully applied in the Portuguese common bean collection used in the present study to identify SNPs and candidate genes associated with fusarium wilt resistance, and photosynthetic response under contrasting well-water and water-deficit conditions [4, 5]. Indeed the mixed origin of the Portuguese germplasm [3] also anticipates the identification of interesting sources of rust resistance both for Andean and Mesoamerican common beans. This is expected since both common bean rust resistance genes and *U. appendiculatus* races can be grouped according to their gene pool of origin and, due to co-evolution, the resistance genes of Andean origin are susceptible to Andean races of the bean rust pathogen but very effective against many Mesoamerican races [33, 34].

The main goal of the present work was to explore the Portuguese common bean natural variation to identify accessions resistant against bean rust, and genes or quantitative trait loci (QTL) controlling rust resistance through a genome-wide association approach. This study will enable the development of molecular tools to increase the breeding efficiency of bean rust resistance.

## Results

### Bean rust infection type and disease severity evaluation

**Fig. 1:**
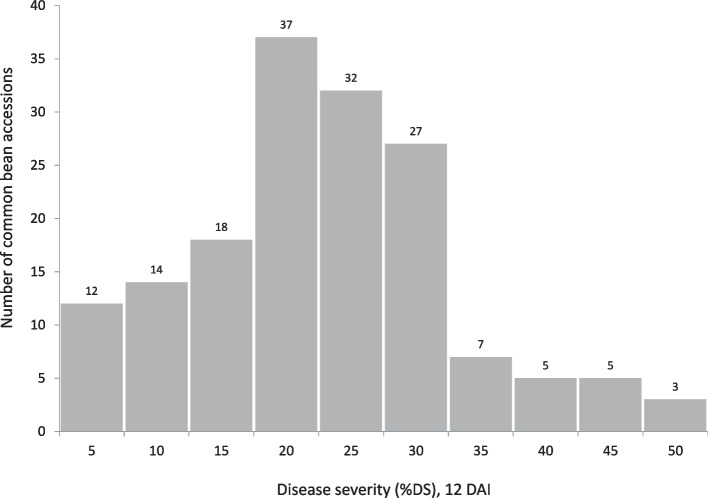
Frequency distribution of bean rust DS adjusted means (BLUEs) caused by *Uromyces appendiculatus* in 160 common bean accessions, 12 days after infection, under growth chamber conditions

The majority of common bean accessions had plants with more than one IT, but the most frequent response to *Uromyces appendiculatus* infection in the Portuguese collection was a compatible interaction (high IT), with no macroscopically visible hypersensitivity. Significant differences were detected among common bean accessions’ DS scores (Wald test *P*-value < 0.001), with the majority of the accessions showing moderate values (20–30%) (Supplementary Table S1 and Fig. 1).

In particular, DS values varied greatly among the accessions displaying high ITs (3 and 4) from 0.8 to 49.3% (Table 1). From these, 20 accessions depicted a reduced DS (≤ 10%), despite a high IT (IT = 3 or 4), and so were considered partially resistant. In addition, eight accessions with both low IT (0/1/2) and DS (≤ 10%), indicative of an incompatible interaction with rust were also identified (SER16, Tio Canela-75, 602, 675, 1644, 2179, 5296, 5391), and considered incomplete hypersensitive resistant (Supplementary Figure S1). We also found accessions showing plants with low IT and, simultaneously, plants with high IT, and these were categorized as mixed-reaction accessions, and removed from further genetic analysis.

**Table 1:** Evaluation of 160 common bean accessions (158 Portuguese, SER16, and Tio Canela-75) 12 days after inoculation with *Uromyces appendiculatus,*, under growth chamber conditions. Accessions were assigned to reaction type categories according to their infection types (IT) and disease severity (not transformed DS, BLUEs range)

Category (no. accessions)	IT^1^	DS (%)	Accessions
Resistant (*n* = 8, 6 PT) (incomplete hypersensitive resistance: low IT and low DS)	0/1/2	0.2 – 8.5	SER16, Tio Canela-75, 602, 675, 1644, 2179, 5296, 5391
Partially resistant (*n* = 20) (high IT and low DS)	3/4	0.5 – 14.1	642, 644, 645, 671, 735, 1653, 2155, 4048, 4071, 4097, 4185, 5286, 5287, 5295, 5300, 5369, 5371, 5372, 5383, Tarrestre
Mixed reactions^2^ (*n* = 16)	0/4	3.4	5297
	1/4	7.5—12.9	632, 4110, 5385
	1/2/4	1.3 – 8.1	1662, 1884, 5376
	1/2/3/4	12.0	648
	2/3/4	8.2	1984
	2/4	9.8 – 15.7	695, 736, 1877, 1911, 1944, 4127, 5381
Susceptible (*n* = 116)	4	15.2– 49.1	116 accessions

^1^Types of infection (IT) present in the plants of each accession

^2^With ITs characteristic of incompatible and compatible interactions in plants of the same accession

**Fig. 2:**
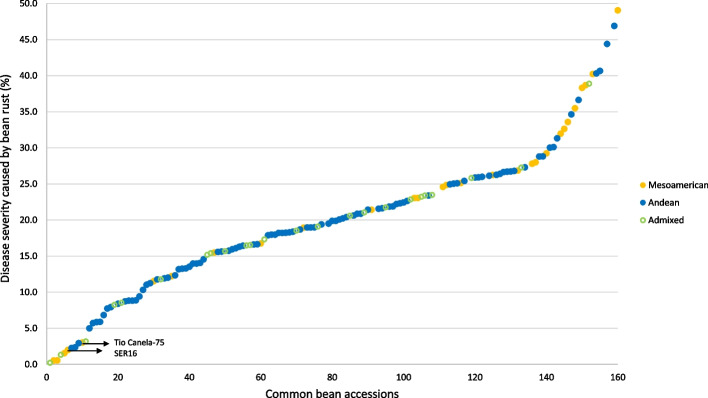
DS adjusted means caused by bean rust in the common bean Portuguese collection, SER16, and Tio Canela-75. The accessions are colored according to the clustering that results from the structure analysis performed together with gene pool representatives [3]. Two groups of accessions were depicted (closed circles), one of Mesoamerican origin (orange) and the other of Andean origin (blue). Open circles (in green) refer to the accessions of admixed origin between gene pools

The eight accessions showing incomplete hypersensitive resistance – simultaneously with IT 0–2 and DS ≤ 10%—were of, Andean (1), Mesoamerican (4, 2 Portuguese accessions plus Tio Canela-75 and SER16), or admixed origin between gene pools (3). On the other hand, 16 of the partially resistant accessions were of Andean origin and two were of Mesoamerican origin (plus two of unknown genetic origin). Among the susceptible accessions, all origins were represented (Fig. 2).

### Analysis of variance components and broad-sense heritability

The Box-Cox transformation of the phenotypic data (DS transf) improved residuals normalization and variance homogeneity. The REML estimators of variance components of the linear model allowed the assessment of the proportion of variance attributed to differences among accessions. DS transf had a high genotypic variance component (σ^2^ accession = 5.443, associated with a high heritability (85.08%) (Table 2).

**Table 2:** Variance components and broad-sense heritability for the bean rust disease severity (DS transf) measured in 158 Portuguese common bean accessions

Trait		Variance components			Heritability (*h*^*2*^) %
	*σ*^*2*^ *accession*	*σ*^*2*^ *block*	*σ*^*2*^ *rep*	*σ*^*2*^ *residual*	
DS transf^1^	5.443	0.952	0.406	2.821	85.08

^1^Box-Cox transformation of DS with λ parameter 0.5

### Genome-wide association mapping for bean rust resistance

From the models tested, the one with a different kinship per chromosome to control the relationship among accessions was the one presenting an inflation value closer to 1 and a Q-Q plot showing fewer *P-*values deviating from the expected uniform distribution that holds under the null hypothesis (Supplementary Figure S2).

**Fig. 3:**
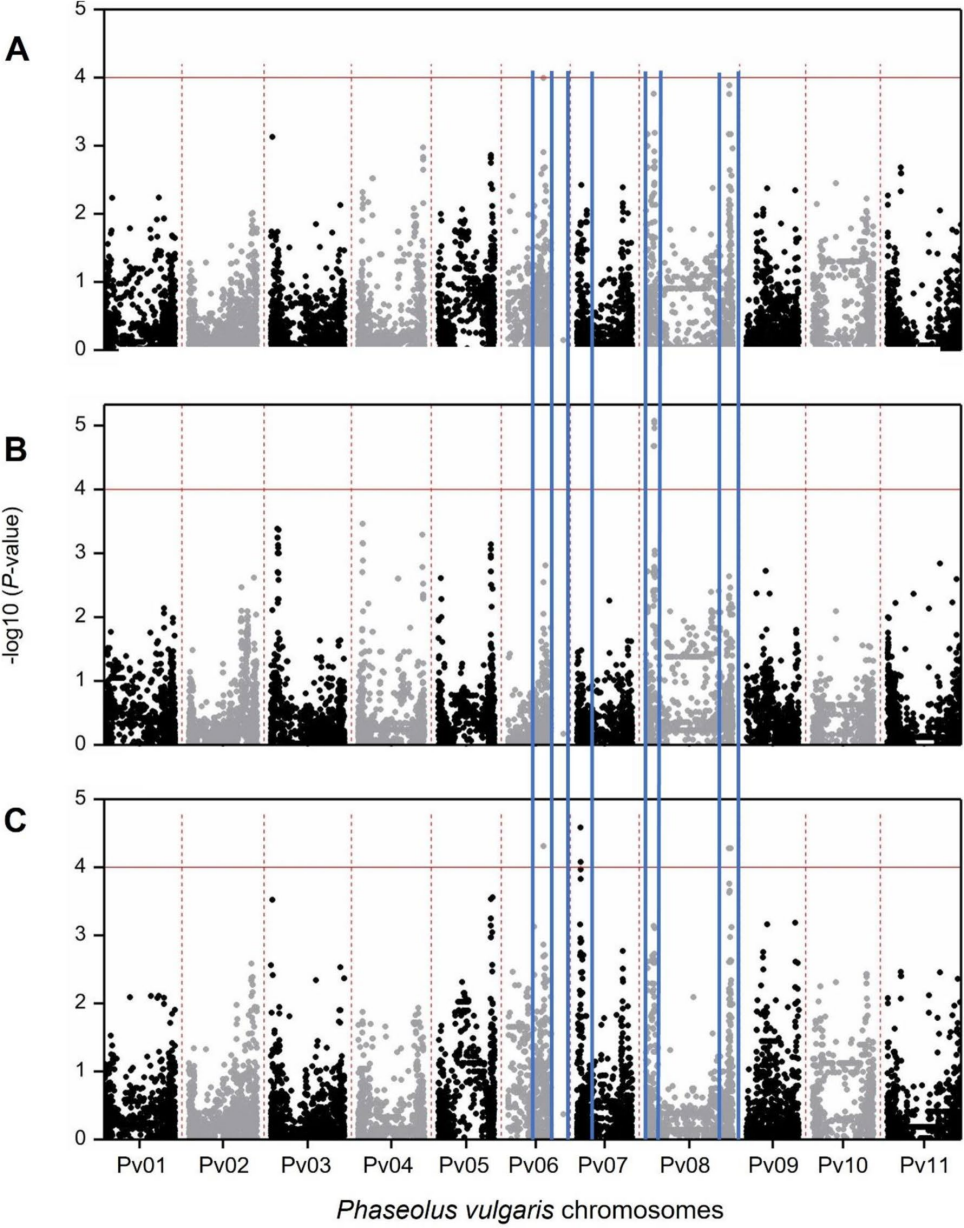
Genome-wide association analysis of bean rust DS in a Portuguese common bean collection. The y-axis represents the -log_10_ (*P-*value) of 9,601 SNPs, and the x-axis shows their chromosomal positions. The horizontal red lines indicate the threshold of significance of -log_10_ (*P-*value) = 4. Vertical blue lines highlight common loci. A – Manhattan plot depicting the marker-trait associations identified with 132 accessions included. B- Manhattan plot depicting the marker-trait associations after removing the 6 accessions considered incomplete hypersensitive resistant, to detect genes responsible for partial resistance. C – Manhattan plot depicting the marker-trait associations after removing the 20 partially resistant accessions, to detect genes responsible for incomplete hypersensitive resistance

Following quality control of SNP data and the exclusion of 16 accessions with mixed reactions, three distinct GWAS were conducted (Fig. 3 and Table 3). In the first GWAS, all the remaining 132 accessions were included and the associations were not strong enough to pass the significance threshold imposed of -log_10_ *P*-value ≥ 4*.* In the second GWAS, the six Portuguese accessions considered incompletely resistant (low IT and low DS) were removed from the first panel, and the remaining 126 were kept to detect genomic regions controlling partial resistance. A total of six SNPs were found significantly associated (-log_10_ *P*-value ≥ 4) with DS transf on chromosome Pv08 (Fig. 3 and Table 3).

**Table 3:** SNP associated (-log_10_ *P*-value ≥ 4) with bean rust disease severity scored 12 days after infection, their position within bean chromosomes, allelic reference and variant for each SNP, the frequency and effect of the variant allele, and the amount of phenotypic variance explained by each SNP associated with DS transf (BLUEs) in the common bean collection. Results are shown for two association mappings after removal of the 16 accessions showing mixed reactions: one with all the remaining accessions except the six considered incomplete hypersensitive resistant (to identify genes controlling partial resistance), and the other with all the remaining accessions except the 20 partially resistant (to detect genes controlling for incomplete hypersensitive resistance). All 11 associations were significant according to the Benjamini-Yekutieli (BY) FDR test

GWAS	Marker name	*Phaseolus vulgaris* chromosome	Position (Mbp)	-log_10_ (*P*-value)	Adjusted *P*-value (BY test)	Allelic reference	Allelic variant	Minor allele frequency	Effect of the allelic variant^a^	V_QTL_ / V_pheno_^b^
Partial Resistance	SNP03379	Pv08	5.580	4.680	0.0001126	G	A	0.210	1.266	0.0580
	SNP03383	Pv08	5.858	4.680	0.0001407	C	T	0.210	1.266	0.0598
	DART07386	Pv08	5.990	5.075	0.0000281	G	C	0.218	1.321	0.0669
	SNP03385	Pv08	6.002	4.680	0.0001689	T	C	0.210	1.266	0.0598
	DART07388	Pv08	6.061	5.040	0.0000563	G	C	0.218	1.315	0.0667
	DART07389	Pv08	6.121	4.961	0.0000844	T	A	0.210	1.295	0.0632
Incomplete hypersensitive resistance	DART05945	Pv06	25.75	4.311	0.0000563	A	T	0.224	2.658	0.1980
	DART06294	Pv07	2.452	4.585	0.0000281	A	T	0.271	-2.184	0.1907
	DART06303	Pv07	2.571	4.078	0.0001407	T	A	0.262	-2.171	0.1869
	SNP03819	Pv08	60.18	4.279	0.0000844	T	C	0.215	2.969	0.2631
	SNP03850	Pv08	61.49	4.279	0.0001126	G	A	0.215	2.969	0.2631

^a^If the effect is positive, an increase in susceptibility is expected, whereas if the effect is negative it is expected an increase in resistance to bean rust

^b^Amount of the variance explained by each association, V_QTL_ = 2freq(1-freq)effect^2^ and V_pheno_ = phenotypic variance of the DS transf (BLUEs)

In the third GWAS performed, the 20 accessions classified as partial resistant accessions were eliminated, and GWAS was performed using the remaining 112 accessions to identify genomic regions controlling incomplete resistance. In this third GWAS, five SNPs were significantly associated (-log_10_ *P*-value ≥ 4) with DS transf on chromosomes Pv06, Pv07, and Pv08. (Fig. 3 and Table 3).

All the associations identified in the second and third GWAS were significant according to the FDR test.

### Proportion of variance explained and SNP effect size

Each association identified between SNP markers and bean rust disease susceptibility (DS) only accounted for a fraction of the observed phenotypic variance within the association panels. These values ranged between 5.8 and 6.7% for the partial resistance GWAS, and between 18.7 and 26.3% for the incomplete hypersensitive resistance GWAS. The variant alleles of SNP03819 and SNP03850, located in chromosome Pv08, were responsible for the highest effect (2.969) and the highest amount of phenotypic variance explained (26.3%) (Table 3).

### SNP favorable allele frequency among common bean gene pool of origin

Bean rust DS varied according to the gene pool of origin of the common bean accessions (*P*-value < 0.001). The comparison of means showed that the accessions with admixed genetic origin between gene pools had lower DS (Table 4).

**Table 4:** Comparison of accessions’ DS transf mean according to their gene pool of origin

Accessions’ gene pool of origin	DS transf mean and Bonferroni test
Admixed	4.945 a
Andean	6.147 b
Mesoamerican	6.207 b

**Fig. 4:**
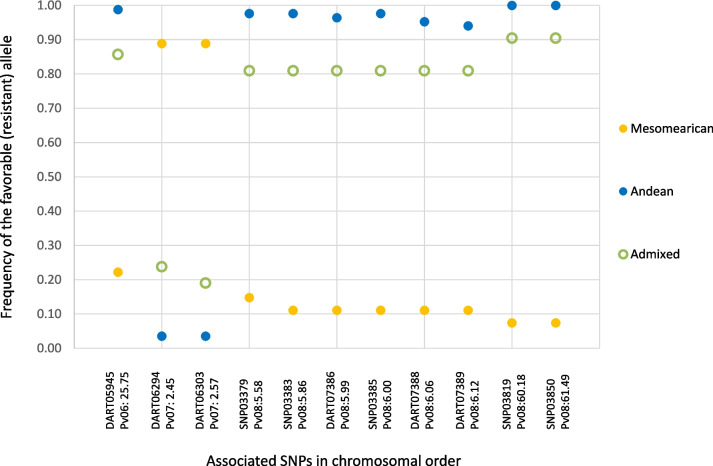
Frequency of the favorable allele (decreasing susceptibility) of the 11 SNPs associated with bean rust DS according to the gene pool of origin of the Portuguese common bean accessions (previously determined in [3]). Each SNP marker is identified in the x-axis by its name and position in the chromosome (in Mbp)

In agreement, the average frequency of the favorable alleles (decreasing susceptibility) on the 11 SNPs associated with bean rust DS also varied according to the gene pool of origin (Fig. 4). For the 9 associations in Pv06 and Pv08 the accessions of Andean and admixed origin had higher frequencies (always > 0.80) of the favorable alleles than the Mesoamerican accessions (always ≤ 0.25). Nevertheless, for DART06294 and DART06303 in Pv07, the accessions of Mesoamerican origin had a higher frequency of the allele conferring resistance than the ones of the Andean and admixed genetic origin.

### SNP favorable allele frequency among disease reaction type categories

**Fig. 5:**
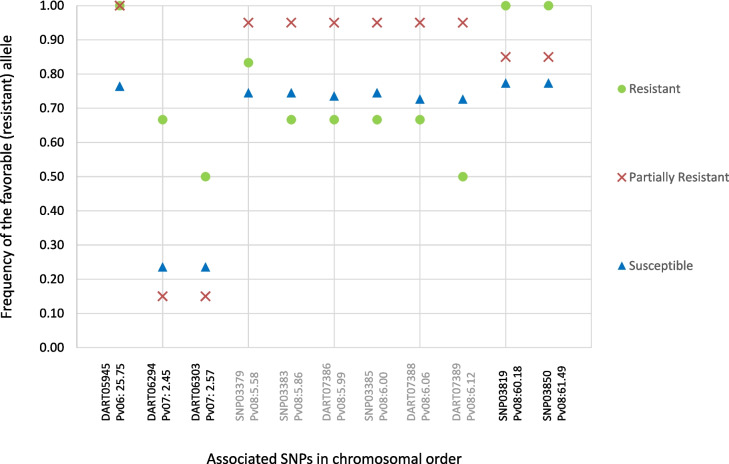
Frequency of the favorable allele (decreasing susceptibility) of the 11 SNPs associated with bean rust DS according to the disease reaction type classes. Each SNP marker is identified in the x-axis by its name and position in the chromosome (in Mbp). The SNPs associated with partial resistance are highlighted in grey in the x-axis, while those associated with incomplete hypersensitive resistance are highlighted in black

As expected, the average frequency of the favorable allele was highest for the partially resistant and for the incomplete hypersensitive resistant accessions in the corresponding association mapping (Fig. 5).

Except for the two associated SNPs in chromosome Pv07, the frequency of the favorable allele was always ≥ 0.50 for all the accessions, regardless of their disease reaction type categorization. This value was ≥ 0.85 for the partially resistant accessions.

Nevertheless, the number of accessions per category varied greatly: as an example, only six accessions are categorized as resistant while 116 are susceptible, which might have influenced this analysis.

Interestingly, two accessions – 602 of admixed origin classified as resistant, and 642 of Andean origin classified as partially resistant – had the favorable (resistant) alleles for all the 11 associated SNPs. These accessions represent promising targets among this Portuguese collection to explore in bean rust resistance breeding.

### Identification of candidate genes associated with bean rust partial and incomplete hypersensitive resistance

The physical positions in the genome of the 11 SNPs significantly associated with bean rust DS were checked using the *JBrowse* tool of *Phytozome* portal and *P. vulgaris* v2.1 genome. The SNP was located within a gene for three out of the six associations with partial resistance and for all the five SNPs associated with incomplete hypersensitive resistance. The functional categorization of the eight genes identified was obtained using MapMan and Mercator online tools (Table 5).

**Table 5:** Genes identified associated with bean rust DS partial resistance and incomplete hypersensitive resistance, their annotation based on the *Phaseolus vulgaris* genome v2.1, and functional categorization according to MapMan/Mercator. NA – not available

GWAS	Marker name	Gene ID	Gene annotation	MapMan/Mercator functional categorization
Partial Resistance	SNP03379	Phvul.008G061600	NA	Not classified
	SNP03383	NA	NA	
	DART07386	Phvul.008G065600	Beta-amylase 7	Enzyme classification. EC_3.2 glycosylase & BZR-type transcription factor
	SNP03385	Phvul.008G065700	Acyl-CoA thioesterase family protein	Not classified
	DART07388	NA	NA	NA
	DART07389	NA	NA	NA
Incomplete hypersensitive resistance	DART05945	Phvul.006G152200	NA	Not classified
	DART06294	Phvul.007G031000	Protein kinase superfamily protein	Enzyme classification. LRK10-1-like protein kinase & EC_2.7 transferase transferring phosphorus-containing group
	DART06303	Phvul.007G032266	Alpha/beta hydrolase	Not classified
	SNP03819	Phvul.008G253400	Eukaryotic aspartyl protease family protein	Enzyme classification. A1-class (Pepsin) protease & EC_3.4 hydrolase acting on peptide bond (peptidase)
	SNP03850	Phvul.008G270700	Co-factor for nitrate, reductase and xanthine dehydrogenase 5 (MOCS3, UBA4, moeB)	Not classified

The candidate genes corresponding to the strongest associations (high -log_10_ (*P*-value)) or with relevant biological functions related to stress resistance were retained for discussion.

## Discussion

The identification of novel sources for bean rust resistance in underused germplasm combined with the study of its genetic basis is very important to develop tools to support common bean durable resistance breeding. We investigated the genetic variation contained in 158 Portuguese common bean accessions, and observed a high diversity in disease response, with DS values ranging from 0.2 to 49.1% and all the ITs present, many times with plants from the same accession showing different ITs. We detected six incomplete hypersensitive resistant accessions (2 of Mesoamerican, 1 Andean, and 3 of admixed origin) and 20 partially resistant accessions (16 of Andean origin, two of Mesoamerican origin, and two of unknown genetic origin to rust. Furthermore, we identified 11 resistance-associated SNPs: six SNPs associated with partial resistance and five with incomplete hypersensitive resistance. In this germplasm, the amount of variance explained by the partial resistance associations varied from 5.8 to 6.7%, whereas it increased to values from 18.7 to 26.3% in the incomplete hypersensitive resistance association. We could have predicted this outcome since incomplete hypersensitive resistance is controlled by major genes with a larger effect on the disease variance. The presence of SNP alleles with high effects in rust DS, together with the high DS heritability (0.85), indicates that improvements can be attained through the combination of these different genomic regions, via selection breeding within the Portuguese common bean germplasm.

The associated SNPs were located in chromosomes Pv06, Pv07, and Pv08, where historical bean rust resistance genes of Andean origin (Ur-4, Ur-9, Ur-12, and Ur-13) have been previously mapped by linkage analysis [17, 35]. Unfortunately, the sparse mapping approach used has hampered a detailed location comparison with the presently identified associated SNPs.

Seventeen percent of the accessions were classified as incomplete hypersensitive (*n* = 6, 4%) and partially resistant (*n* = 20, 13%), and are preferential targets to be used in rust resistance breeding. In particular, two accessions – 602 of admixed origin classified as incomplete hypersensitive resistant, and 642 of Andean origin classified as partially resistant – had the favorable alleles for all the 11 associated SNPs and are now privileged target sources of resistance.

Except for the two associated SNPs located in Pv07, the frequency of the favorable allele was ≥ 0.85 for the partially resistant accessions. Also, and as expected, for the SNPs associated with incomplete hypersensitive resistance, the frequency of the favorable allele was highest in the six accessions showing this type of resistance mechanism. Interestingly, the favorable alleles of the two SNPs in Pv07 were mainly present in accessions of Mesoamerican origin.

The continuous distribution of DS scores and the observed diversity of plants' responses to rust infection supported the existence of an oligogenic base in the Portuguese common bean response to this *U. appendiculatus* f. sp. *phaseoli* isolate. This was confirmed by the subsequent association mapping results. Previous studies on common bean response to rust infection described several types of resistance – a predominance of dominant monogenic, but with some oligogenic in both Andean and Mesoamerican lines—and reported their use in breeding [17, 36]. However, Hurtado-Gonzales et al. [20] showed that *Ur-3*, *Ur-4*, *Ur-5*, *Ur-6*, and *Ur-7* have been overcome by many *U. appendiculatus* races, especially the ones of Mesoamerican origin. But on the other hand, the resistance conferred by the Mesoamerican genes *Ur-3* + and *Ur-14*, whose sources were the genotypes Mexico 325 and Ouro Negro, kept effective against the tested races [17, 20, 37]. The development of genotypes with multiple genes stacked, both major and minor, for bean rust resistance from different gene pools of origins is thus an effective strategy for durable resistance. It becomes more difficult for the pathogen to overcome the resistance conferred by several genes [19, 38], creating durable resistance to the multiple races of rust pathogen [23, 25]. Furthermore, we observed that the DS average value was lower for the accessions of admixed origin than for the accessions of Andean and Mesoamerican origin. This suggests that the accessions of admixed origin might have a different combination of alleles more beneficial to respond to rust.

It is well-known that the coevolution between the gene pools of the host and the pathogen gave rise to isolates that are virulent only in Andean or Mesoamerican germplasms [39, 40]. Accordingly, the average frequency of the favorable allele of associated SNPs varied between the two gene pool of origin. We observed that, for most associations, the accessions of Andean and admixed origin had higher frequency of the allele conferring resistance to bean rust than the accessions of Mesoamerican origin. Nevertheless, it is necessary to have in mind that, in this association panel, the accessions of Mesoamerican origin are in smaller number in comparison to those of Andean origin (27 versus 96). Yet, this suggests that the resistance of the accessions of Mesoamerican origin can be improved by introgression of favorable alleles from these Andean and admixed origin accessions into Mesoamerican germplasm. Still, we identified two incomplete hypersensitive resistant Portuguese accessions of Mesoamerican origin – 1644 and 2179 that could the other way around be applied in resistance breeding of Andean germplasm, and in this way contributing to different common bean commercial classes breeding programs.

We were able to identify potential candidate genes for eight out of 11 resistance-associated SNPs, which corresponds to a resolution to the gene level of 73%. Phvul.008G065600, which encoded a β-amylase 7, was the gene found underlying the association with the highest -log_10_ (*P*-value) and effect in DS variation in the panel used to identify partial resistance. In *Arabidopsis thaliana*, β-amylases are described to have a role in starch degradation, and a reduced carbohydrate availability influences plant susceptibility in interactions with hemibiotrophic and biotrophic fungal pathogens [41]. In chickpeas, β-amylase expression levels were induced in resistant interactions against fusarium wilt, another fungal disease [42]. Phvul.008G065700 was another gene found associated with partial resistance. This gene encoded an acyl-CoA thioesterase family protein involved in the lipid metabolism that hydrolyzes thioester bonds in acyl-CoA metabolites. It is known that plant lipids act as signal transducers in signaling pathways essential to plants’ response to pathogen attacks [43].

On the other hand, the SNP associated with the highest -log_10_ (*P*-value) and effect in DS variation in the association mapping to identify genes related to hypersensitive response was within the gene Phvul.007G031000, which encodes a protein kinase superfamily protein. Protein kinases are important for the perception and transduction of signals during pattern-triggered and induce effector-triggered immunity in plant-pathogen interactions [44]. In common bean pathosystems, it was reported their role in regulating the plant resistance against fungal diseases such as anthracnose, caused by *Colletotrichum lindemuthianum,* and fusarium wilt, caused by *Fusarium oxysporum* f. sp. *phaseoli*, and against common bacterial blight caused by *Xanthomonas phaseoli* pv. *phaseoli* [45–47]. Also, with a recognized role in plant-pathogen interactions, we identified the gene Phvul.008G253400 encoding for an aspartyl protease. Aspartyl proteases are an integral part of plant defense systems, with several hubs of action, from pathogen recognition and priming to the activation of plant hypersensitive response [48].

All the potential candidate genes described above need further studies to be validated by functional studies in resistant and susceptible accessions, in different genetic backgrounds, or in segregating biparental populations. Additionally, it would be interesting to test other rust isolates and races to verify the broadness or specificity of the resistance sources here identified.

Due to the experimental design and growth chamber space restrictions, the size of the collection analyzed was limited. Further, we decided to remove from the association panel some accessions with mixed IT types, probably due to residual levels of heterozygosity, to avoid this confounding factor in the GWAS. Despite that, the reasonably high heritability of the bean rust DS compensates for the relatively small population size, since the power for detecting QTLs depends not only in the size of the association panel but also on the heritability of the trait [49]. Moreover, the more precise and specific phenotyping carried out in this study allowed the discrimination between genomic regions associated with partial resistance and incomplete hypersensitive resistance. This GWAS approach might be also applied to other plant-pathogen systems with identical disease assessment. It was possible to pinpoint candidate genes for all the associations identified, and this strengthened and validated the usefulness of the common bean association panel used. The variety of IT identified in the present study, reflected the diversity of resistance mechanisms deployed by the Portuguese germplasm.

This study is a significant contribution to understanding the genetic basis of bean rust resistance, using the natural diversity present in underutilized Portuguese common bean accessions. We found new sources of partial and incomplete hypersensitivity resistance to bean rust, controlled by multiple genes. These resistant alleles, acting in the control of bean rust resistance through different mechanisms, can facilitate the transfer of durable resistance to high-yielding cultivars. This transfer can be achieved through precise breeding methods, incorporating markers to assist in the selection of beans resistant to rust.

## Conclusions

In this study, we identified new sources of resistance to common bean rust (*Uromyces appendiculatus*) in a representative collection of the Portuguese germplasm, known to have accessions with an admixed genetic background between the original Mesoamerican and Andean gene pools.

Common bean revealed an oligogenic control of resistance to rust with 11 SNPs associated with partial and incomplete hypersensitive resistance detected in chromosomes Pv06, Pv07, and Pv08. Candidate resistance genes were proposed for eight of the associations. They included several resistance-associated enzymes, namely β-amylase 7, acyl-CoA thioesterase, protein kinase, and aspartyl protease. This research provides promising genomics targets to develop functional molecular tools to support bean rust resistance precision breeding.

## Methods

### Phenotypic data

#### Plant material and growing conditions

The 158 Portuguese common bean accessions used in this study are part of the national common bean collection held at the Portuguese Plant Germplasm Bank (BPGV, INIAV, Braga, Portugal, https://www.iniav.pt/bpgv/) (Supplementary Table S1). Prior to the present study, the collection was multiplied under controlled conditions for two years and molecularly and morphologically characterized [3]. On one hand, the molecular diversity of this collection was assessed by using 10 individuals per accession screened with 21 microsatellites and a DNA marker for phaseolin-type [3]. Then, 14 traits related to seed and plant morphology were also characterized [3]. On average, the collection had an observed heterozygosity of 0.027. Also in this previous study, we molecularly compared the Portuguese accessions with 17 wild relatives and representative accessions from the Andean and Mesoamerican gene pools. This allowed the assignment of each Portuguese accession to its gene pools of origin, and their distribution into three primary clusters via structure analysis. Structure analysis enabled to conclude that the majority of the accessions are of Andean origin. Still, one-third showed admixed genetic, probably being the result of Andean x Mesoamerica gene pools hybridization [3].

In this study, besides the 158 Portuguese accessions, we evaluated two Mesoamerican breeding lines – SER16 and Tio Canela-75 –, gently provided by the International Center for Tropical Agriculture (CIAT, Colombia), as standards. SER16 is a drought-resistant line [50], and Tio Canela-75 is a commercial variety described to be resistant to abiotic and biotic stresses, including bean rust [51]. All seeds were surface-sterilized using a 20% solution of sodium hypochlorite and an exposure time of 20 min, followed by rising two times with sterile water for 20 min. Next, seeds were sown in 6 × 6x8 cm plastic pots filled with a mixture of peat and sand [3:1 (v/v)]. Three to five plants per common bean accession and inoculation were used, with one seed per pot. Pots were placed in a plant growth chamber kept at 27 ± 2 °C under a photoperiod of 14 h light (~ 250 μmol.m^−2^ s^−1^) and 10 h dark, and with a relative humidity of ~ 60%.

### Experimental design

The collection of 160 common bean accessions (158 Portuguese, plus the standards SER16, and Tio Canela-75) was evaluated against bean rust and divided into four independent experiments due to growth chamber space constraints. To account for the experimental effect (Block), five accessions were repeatedly evaluated in all the experiments following a complete block design. The remaining genotypes followed an incomplete block design.

### Fungal isolate and inoculation

Urediniospores from *Uromyces appendiculatus* (*Pers*.) Unger were collected on common bean plants in Lanestosa, Vizcaya, Spain, and stored at -80 °C. Plants from susceptible accessions (cv ‘Xana’, ‘BAT93’, and the commercial variety Hostal) were used to multiply and obtain fresh spores before the experiment. The experiment followed a randomized design. Two-week-old plants were inoculated using a simple hand-operated duster. Three independent and consecutive inoculations were performed, each one with three to five plants per accession. The inoculum density was defined as 30 spores.mm^−2^, achieved by dusting 2 mg of *U. appendiculatus* spores per seedling, diluted (1:10) in pure talcum powder. Plants were first placed in an incubator for 24 h at 20 °C in complete darkness and 100% relative humidity, and then relocated to the initial growth chamber.

### Disease assessment

The infection type (IT) was scored 12 days after inoculation (DAI) using the IT scale from Stakman et al. [52]. In this 0–4 scale, 0 stands for immune—no symptoms, 1 very resistant—hypersensitive necrotic spots with minute pustules little sporulating, 2 moderately resistant—necrotic halo surrounding small pustules, 3 moderately susceptible—chlorotic halo surrounding pustules, and 4 susceptible—well-formed pustules, without chlorosis or necrosis. “;” indicates the existence of necrotic spots. Two ITs separated by a slash “/”, e.g., 3/4, indicates that both ITs are present in equal proportion in the same plant, while one IT followed by another in parenthesis, e.g., 3(4), indicates that both IT scores are present in the leaves from the same plant, but the second IT is less frequent.

Disease severity (DS) was evaluated on the cotyledonary leaves as the percentage of leaf area covered by rust pustules 12 DAI. Accessions were classified as resistant based on hypersensitivity when IT = 0–2 and DS ≤ 10% (complete hypersensitive resistant when IT = 1 and DS = 0, incomplete hypersensitive resistant otherwise), partial resistant when IT = 3–4 and DS ≤ 10%, and susceptible when IT = 3–4 and DS > 10%.

### Phenotypic data analysis

Quality control of phenotypic data (DS) was performed by assessing the normal distribution of residuals in the quantile–quantile (Q–Q) plot, homogeneity of variance, and the presence of outliers. A Box-Cox transformation was applied to DS values (DS transf) to homogenize their variance so that the residuals are normally distributed.

The restricted maximum likelihood (REML) algorithm was used to estimate the variance components of the linear mixed model applied: *Trait (DS transf)* = *block* + *accession* + *accession.rep* + *error*. In this model, *accession* is the genotypic term, *block* is the term that identifies the four experiments needed to have all the 160 accessions evaluated, and *accession.rep* corresponds to the interaction between each accession and the three independent inoculations performed. To estimate broad-sense heritability, variance components were obtained for each random term. In a second step, *accession* term was fitted as fixed to obtain the linear unbiased estimates (BLUEs) of DS transf for each accession. These BLUEs were the input used as phenotypic values in the association mapping analysis. Additionally, a second model was applied adding the accessions’ gene pool of origin (*gene pool*) as a fixed term: *Trait (DS transf)* = *gene pool* + *accession* + *accession.rep.*

The significance of the fixed terms was assessed using a Wald test. Additionally, a comparison of DS transf means among each gene pool was performed using a Bonferroni test and *P*-value ≤ 0.05. All this statistical data analysis was performed using the Genstat software, 22nd edition [53].

### Genotypic data

#### Association mapping analysis

The genotypic dataset used previously in [4] was retrieved for the present association analysis and consists of 9,601 SNP markers from the Illumina Infinium BARCBean6K_3 BeadChipTM assay and DArTseq™ analysis. In the case of more than 25% of missing data and when the minor allele frequency was less than 0.05, the SNPs were removed. The same occurred for the accessions with more than 25% of genotypic missing data and for the ones showing mixed reactions.

Genome-wide association studies were performed using the BLUEs values of DS transf and the QTL menu from the Genstat software. Three independent GWAS were performed. In the first one, we kept all the accessions that passed the quality control. In a second analysis, we eliminated the incomplete resistant accessions, i.e., with low DS and low IT, with the objective of detecting genomic regions controlling partial resistance. In this way, we avoided covering the effect of minor genes responsible for partial resistance. Following the same reasoning, in the third analysis, the accessions classified as partial resistant, with high IT and low DS were eliminated from the analysis, to detect the genomic regions controlling incomplete hypersensitive resistance.

All these GWAS were performed fitting markers as fixed and accessions as random terms on REML [54]. Four models were tested to detect marker-trait associations: a null or naïve model [Phenotype = SNP + Error], not taking into account the population structure or family relatedness; a model controlling for population structure (Q), using 15 principal components [Phenotype = Q + SNP + Error]; and two models controlling for familial relatedness (K), one with accession random effects accounting in a single kinship matrix K [54] [Phenotype = SNP + Accession + Error], and the other using a different kinship matrix for each chromosome, calculated only taking into consideration the SNPs located on the other 10 chromosomes [55]. To perform this last procedure, the kin function of R package synbreed [56] and the VanRaden measure were applied [57].

The inspection of the inflation factor and Q-Q plot of *P*-values for each model is an indication of what extent the model corrected for genetic structure/relatedness among accessions, therefore avoiding false positives.

The *P-value* of each SNP associated test was plotted versus its position in the chromosome. The threshold of − log_10_ (*P-*value) was set as 4, and significant marker-trait associations were identified. Considering the association panel size and observing the background noise in the Manhattan plots, this threshold seems appropriate to identify truly associated SNPs and potentially interesting genomic regions, which would be missed, for instance, by the very stringent Bonferroni-corrected threshold of significance. Besides, to control type I errors due to multiple testing, adjusted *P*-values following the Benjamini and Yekutieli false discovery rate (FDR) method [58] were calculated, considering α = 0.1 and setting the actual number of independent tests as the number of LD blocks in the genome (K = 520).

The effect of the SNP with the minor allele frequency was calculated for the significant associations within each association panel tested. Using the formula V_QTL_/V_pheno_ [59], in which V_QTL_ = 2freq(1-freq)effect^2^ and V_pheno_ is the phenotypic variance of the DS transf adjusted means for each association panel, the amount of variance explained by each SNP-trait association was calculated. The correlation between the allele frequency, the gene pool of origin, and the resistance classification of each accession [3] was also examined.

#### Candidate genes identification

A local linkage disequilibrium (LD) inspection was performed to define chromosomal regions to search for candidate genes. A gene was examined if it carried an associated SNP or was in LD with a SNP associated with DS transf adjusted means. LD was previously calculated for each chromosome using the squared coefficient of correlation between marker pairs*,* r^2^ [4]. To assess the presence of nearby SNP markers linked to the trait, a genomic area surrounding the associated SNPs was determined based on a stringent LD decay threshold (r2 > 0.2). Subsequently, we searched within each LD block for potential candidate genes using the JBrowse tool in *Phaseolus vulgaris* v2.1 genome, available in Phytozome v13 portal (DOE-JGI and USDA-NIFA, http://phytozome.jgi.doe.gov/). Candidate genes annotation was obtained from the file “Pvulgaris_442_v2.1.annotation_info.txt, also in Phytozome v13. Identifiers from *KEGG* and *PANTHER* databases were used to retrieve information on the pathways involved and the functions of the candidate genes. In addition, candidate genes were assigned to MapMan functional categories, using Mercator4 v4.0 [60].

### Supplementary Information

**Additional file 1:** **Table S1.** Infection type and % of disease severity (DS) of 160 common bean accessions evaluated for resistance against *Uromyces appendiculatus *infection, under growth chamber conditions, 12 days after inoculation. The accessions are all Portuguese, with the exception of SER16 and Tio Canela-75, two lines of Mesoamerican origin used as a reference for comparative purposes. **Figure S1.** Examples of incomplete hypersensitive resistance (A) and partial resistance (B). **Figure S2.** Quantile-quantile (QQ) plots corresponding to the association mapping analyses using a linear mixed model with one kinship matrix per chromosome to account for the genetic relatedness among accessions. A – association panel with all the 132 common bean accessions. B – association panel after removing the 6 accessions considered incomplete hypersensitive resistant, to detect genes responsible for partial resistance. C – association panel after removing the 20 partially resistant accessions, to detect genes responsible for incomplete hypersensitive resistance.

## Data Availability

All the data that supports the findings of this study is available in the supplementary materials. The raw data is available from the corresponding author on reasonable request.

## Abbreviations

BLUE: Best linear unbiased estimate
BY: Benjamini-Yekutieli
DAI: Days after inoculation
DS: Disease severity
FDR: False discovery rate
GWAS: Genome-wide association study
IT: Infection type
LD: Linkage disequilibrium
Q-Q: Quantile–quantile
REML: Restricted maximum likelihood
SNP: Single nucleotide polymorphism
