# Supplementary material for: Identification of novel sources of partial and incomplete hypersensitive resistance to rust and associated genomic regions in common bean
Source: BMC Plant Biol. 2023 Dec 1;23:610. doi: 10.1186/s12870-023-04619-8 (PMC10691055; doi:10.1186/s12870-023-04619-8)
Supplement: Supplementary file 1 — Additional file 1: Table S1. Infection type and % of disease severity (DS) of 160 common bean accessions evaluated for resistance against Uromyces appendiculatus infection, under growth chamber conditions, 12 days after inoculation. The accessions are all Portuguese, with the exception of SER16 and Tio Canela-75, two lines of Mesoamerican origin used as a reference for comparative purposes. Figure S1. Examples of incomplete hypersensitive resistance (A) and partial resistance (B). Figure S2. Quantile-quantile (QQ) plots corresponding to the association mapping analyses using a linear mixed model with one kinship matrix per chromosome to account for the genetic relatedness among accessions. A – association panel with all the 132 common bean accessions. B – association panel after removing the 6 accessions considered incomplete hypersensitive resistant, to detect genes responsible for partial resistance. C – association panel after removing the 20 partially resistant accessions, to detect genes responsible for incomplete hypersensitive resistance. [file 12870_2023_4619_MOESM1_ESM.pdf]

# Identification of novel sources of partial and incomplete hypersensitive resistance to rust and associated genomic regions in common bean

**Authors**

Susana T. Leitão[1*], Diego Rubiales[2] and Maria Carlota Vaz Patto[1]

[1]ITQB NOVA, Oeiras, Portugal

[2]Institute for Sustainable Agriculture-CSIC, Córdoba, Spain

*Correspondence: sleitao@itqb.unl

## Supplementary Material

**Table S1:** Infection type and % of disease severity (DS) of 160 common bean accessions evaluated for resistance against *Uromyces appendiculatus* infection, under growth chamber conditions, 12 days after inoculation. The accessions are all Portuguese, with the exception of SER16 and Tio Canela-75, two lines of Mesoamerican origin used as a reference for comparative purposes.

| Accession nr (INIAV-Oeiras PRT005 collection)[1] | Deposition accession nr (INIAV – BPGV database)[1] | Gene pool of origin[2] | Infection type[3] | DS range | DS (BLUEs) |
|---|---|---|---|---|---|
| 579 | BPGV34894 | Mixed | ;/4 | 1 - 20 | 15 |
| 583 | BPGV34895 | Mixed | 2/4, 4, ;(4), ;/4 | 10-50 | 23 |
| 584 | BPGV34896 | Mixed | 2/4, 4 , ;/4 | 2-60 | 26 |
| 587 | BPGV34897 | Andean | ;(4)/4 , ;/4 , ;/3/4 | 5-25 | 19 |
| 592 | BPGV34898 | Andean | 2/4 , ;/4 | 5-40 | 17 |
| 600 | BPGV34899 | Mixed | 4 , ;/4 , 3/4 | 5-20 | 19 |
| 601 | BPGV34900 | Andean | ;/4 , ;(4) , ;/3/4 | <1-20 | 18 |
| 602 | Not yet attributed | Mixed | ;/2 | <1-5 | 9 |
| 610 | BPGV34901 | - | ;/4 , ;/2/4 | 10-30 | 26 |
| 620 | BPGV34902 | Andean | ;/4 , ;/3/4 , 3/4 | 2-30 | 25 |
| 621 | BPGV34903 | Andean | ;/4 | 10-20 | 22 |

| | | | | | |
|---|---|---|---|---|---|
| 623 | BPGV34904 | Mesoamerican | ;/4 , 3/4 , 4 , ;/3/4 | 10-25 | 25 |
| 632 | BPGV34905 | Andean | ;/4, ;(4), 4, ;/1(4), ;/1/4 | <1-25 | 8 |
| 633 | BPGV34906 | Andean | ;/4 , 4 | 5-15 | 16 |
| 635 | BPGV34907 | Andean | ;/4 , 3/4 , 4 | 15-30 | 31 |
| 638 | BPGV21937 | - | ;/4 , 4 , 3/4 | 10-35 | 26 |
| 639 | BPGV34908 | Mixed | ;/4 , 4 | 5-15 | 17 |
| 642 | BPGV34909 | Andean | ;/4 , ;(4) | <1-15 | 13 |
| 644 | BPGV34910 | Andean | 4, 4(;), ;/4, 4 | <1-10 | 5 |
| 645 | BPGV34911 | Andean | 4 | <1 - 45 | 13 |
| 648 | BPGV34912 | Andean | ;/4, ;/2/4, ;/4(3), ;/1/2, ;/2, 3/4, 4 | <1-50 | 12 |
| 654 | BPGV34913 | Mesoamerican | 4, ;/4, 4(3), 3/4, 3(4) | 20-80 | 34 |
| 667 | BPGV34914 | Andean | 3(4) , 4 | 5-45 | 18 |
| 670 | BPGV34915 | Andean | 3/4, 4, 3(4), 4(3) | 5-70 | 27 |
| 671 | BPGV34916 | Andean | ;/4 , ;(4) , 4 | 1-20 | 14 |
| 675 | BPGV34917 | Mixed | 2/;(2) , ;/2 | 1-5 | 8 |
| 677 | BPGV21963 | - | 4 | 20-30 | 27 |
| 695 | BPGV34918 | Andean | ;/4 , 2/;(4), 4, ;/2/4 | 1-10 | 10 |
| 698 | BPGV34919 | Andean | 3/4, 4 | 5-60 | 18 |
| 700 | BPGV34920 | Andean | ;/4, ;/3/4 | 1-25 | 16 |
| 706 | BPGV34921 | Andean | 4 | 20-55 | 27 |
| 735 | BPGV34922 | Andean | ;(4) , ;/4, 4, 3/4 | <1-20 | 4 |
| 736 | BPGV34923 | Andean | ;/4 , ;(4) , ;/2/4 | <1-15 | 9 |
| 737 | BPGV34924 | Andean | ;/4, 4, 3/4, 3(4), 4(3), 3 , 4 | 5-60 | 27 |
| 747 | BPGV34925 | Andean | ;/4, ;(4), 4, 3/4 | 1-60 | 19 |
| 748 | BPGV34926 | Mesoamerican | 4 , ;/4 , ;/3/4 , 3/4 | 5-40 | 25 |
| 1631 | BPGV34928 | Andean | 4 | 10-50 | 16 |
| 1636 | BPGV34929 | Mesoamerican | 4(3) , ;/4(3) , 4(;) , 3/4, , 4 , ;/3/4, 3, 4, ;/4 | 15-70 | 40 |
| 1644 | BPGV34930 | Mesoamerican | ;, ;/1, ;/2, ;(2), 1/2, ;/1/2, 2 | < 1 - 5 | 1 |
| 1651 | BPGV34931 | Mesoamerican | ;/4 , ;/3/4 , 3/4 , ; , 4 | 5-40 | 29 |

| | | | | | |
|---|---|---|---|---|---|
| 1653 | BPGV34932 | Andean | 4 | 2-40 | 14 |
| 1654 | BPGV34933 | Andean | ;/4 | 1-20 | 16 |
| 1662 | BPGV34934 | Andean | ;/1 , ;(4)/2 , ;/2 , ;/2/4 , ;(4) | 1-15 | 9 |
| 1663 | BPGV34935 | Andean | 3/4 , 4 , 4(3) , 4/3 | 15-50 | 30 |
| 1867 | BPGV34936 | Mesoamerican | ;/4 , ;/3/4 , 3/4 | 15-40 | 28 |
| 1871 | BPGV34937 | Andean | ;/4 , ;(4) , ;/3/4 , 3/4 | <1-25 | 21 |
| 1877 | BPGV22231 | Andean | ;/4 , ;/2/4 , 4 | 5-15 | 15 |
| 1883 | BPGV34938 | Andean | ;/4 | 5-20 | 16 |
| 1884 | BPGV22237 | Andean | 4 , ;/4 , ; /2/4 , ;/1, ;/1/4 | 2-15 | 9 |
| 1889 | BPGV22242 | Andean | ;/4, 3/4, 4 | 15-25 | 25 |
| 1892 | BPGV22245 | Mesoamerican | ;/4 , ;/3/4 | <1-30 | 21 |
| 1893 | BPGV22246 | Andean | 4 , ;/4 | 10-20 | 20 |
| 1897 | BPGV22249 | Mesoamerican | ;/4 , 3/4 | 10-30 | 26 |
| 1911 | BPGV34940 | Andean | ;/4 , 2/;(4) , ;/2/4 | <1-15 | 11 |
| 1917 | BPGV34941 | Andean | ;/4 , ;/2/4 | 10-20 | 18 |
| 1918 | BPGV34942 | Mesoamerican | 3/4 , 4, ;/4 | 10-30 | 25 |
| 1926 | BPGV34943 | Andean | ;/4 , 4 , ;(4) | 1-20 | 18 |
| 1927 | BPGV34944 | Andean | ;/4 , 4 | 1-40 | 22 |
| 1932 | BPGV34945 | Mesoamerican | ;/4 , ;/3/4, 4 , 3/4 , ;/4(3) | 1-40 | 23 |
| 1933 | BPGV34946 | Andean | ;/4, 4 | 10-20 | 20 |
| 1937 | BPGV34947 | Andean | 4 , ;/4 | 15-40 | 35 |
| 1938 | BPGV34948 | Mesoamerican | 4 , ;/4 , ;/3/4 , 3/4 | 10-40 | 27 |
| 1943 | BPGV22284 | Andean | ;/4 , 4 | 15-25 | 26 |
| 1944 | BPGV34950 | Andean | ;/4 , ;/2/4 | 5-15 | 13 |
| 1948 | BPGV34952 | Mixed | ;/4 , 3/4 , 4 | 1-30 | 21 |
| 1952 | BPGV34953 | Mesoamerican | 4 , ;/4 , ;/3/4 | 10-40 | 32 |
| 1955 | BPGV34954 | Mesoamerican | 3, 4, 3(4), 3/4, ;/4 | 15-65 | 39 |
| 1956 | BPGV34955 | Mixed | 4 , ;/4 , ;/3/4, 3/4 | 10-30 | 23 |
| 1961 | BPGV34956 | Andean | ;/4 , 4 , 3/4 , ;/3/4 | 10-30 | 27 |
| 1964 | BPGV34958 | Mesoamerican | 4 , 3/4 , ;/3/4 | 15-40 | 33 |
| 1966 | BPGV34959 | Andean | ;/4, 4 | 5-35 | 20 |

| | | | | | |
|---|---|---|---|---|---|
| 1975 | BPGV34960 | Andean | ;(1)/4 , 4 | 5-20 | 18 |
| 1976 | BPGV34961 | Andean | 4 | <1 - 50 | 22 |
| 1979 | BPGV34962 | Mesoamerican | 4, ;/4, 3/4, ;/3/4 | 5-40 | 28 |
| 1984 | BPGV34963 | Andean | 2, 3(2), 3(4), ;(4), 4 | 1-30 | 8 |
| 2081 | BPGV22404 | Andean | ;/3/4, 3/4, 4, ;/4 | 2-50 | 19 |
| 2126 | BPGV22439 | Andean | 3/4, 4 | 10-80 | 40 |
| 2155 | BPGV22466 | Andean | 3, 4 | 5-20 | 8 |
| 2159 | BPGV22470 | Mixed | 3/4, 4, ;/4, 4(3) | 15-80 | 39 |
| 2179 | BPGV22486 | Mesoamerican | 0 , ; , ;/1 , ;(1), 1 | 0-5 | 2 |
| 2189 | BPGV34964 | Andean | ;/4 , ;/2/4 | 5-20 | 18 |
| 2192 | BPGV22494 | Andean | ;/4 , ;/3/4 , 3/4 | 10-25 | 22 |
| 4038 | BPGV34965 | Andean | ;/4 , 4 , 3/4 , ;/3/4 | 10-20 | 22 |
| 4044 | BPGV22504 | Andean | 4 , ;/4 , ;/3/4, 3/4 | 15-30 | 26 |
| 4048 | BPGV22505 | Andean | ;, 4, ;/4 | <1 - 40 | 9 |
| 4049 | BPGV22506 | Andean | 3/4, 4 | 5-45 | 22 |
| 4050 | BPGV22507 | Andean | 3, 3/4, 4 | 2-60 | 23 |
| 4051 | BPGV22508 | Andean | 3, 3/4, 4 | 1-60 | 21 |
| 4064 | BPGV22513 | - | ; / 4 | 5-20 | 19 |
| 4067 | BPGV34966 | Andean | ;/4 | 5-20 | 19 |
| 4070 | BPGV22514 | Andean | 3, 3/4, 4 | 5-45 | 22 |
| 4071 | BPGV22515 | Andean | 4 | <1 - 15 | 3 |
| 4072 | BPGV22516 | Andean | 4, 4(3) | 2-50 | 16 |
| 4073 | BPGV22517 | Mesoamerican | 4, 3/4, 4(3) | 30-80 | 49 |
| 4074 | BPGV22518 | - | ;/4 , ;/3/4, 4 | 5-25 | 21 |
| 4081 | BPGV34967 | Mixed | 3/4, 4 | 8-60 | 27 |
| 4085 | BPGV22521 | Mixed | 4 | 5-40 | 19 |
| 4088 | BPGV22523 | Andean | ;/3/4 , 3/4 , 4 | 10-25 | 19 |
| 4097 | BPGV22524 | Andean | 3/4 | 2-25 | 6 |
| 4099 | BPGV22525 | - | 3/4, 4(3), 4 | 20-80 | 41 |
| 4100 | BPGV22526 | Andean | 3(4), 4 | 10-40 | 19 |
| 4108 | BPGV22528 | Andean | ;/4 , 4 | 10-30 | 21 |

| | | | | | |
|---|---|---|---|---|---|
| 4110 | BPGV34968 | Mixed | ;/1 , ;/4 | 1-10 | 12 |
| 4119 | BPGV22530 | Andean | 3, 3/4, 4 | 4-80 | 41 |
| 4120 | BPGV22531 | Andean | 3, 3/4, 4 | 5-60 | 21 |
| 4127 | BPGV22533 | Andean | 2(4), 2/4 | 8-30 | 12 |
| 4133 | BPGV34969 | Andean | ;/4, 4, 3/4 | 15-50 | 26 |
| 4135 | BPGV22535 | Mesoamerican | 3/4, 4, ; , ;(4) | 1-45 | 19 |
| 4144 | BPGV22538 | Mesoamerican | ;/4 , 4 | 5-25 | 17 |
| 4149 | BPGV34970 | Andean | 4(;), ;/4 , 4 , 3/4 | 10-40 | 29 |
| 4150 | BPGV22540 | Andean | 3, 4, 3/4, 4(3) | 8-60 | 26 |
| 4162 | BPGV22545 | Mixed | ;(4) , ;/4; 4 , 3/4 , ;/3/4 | 5-30 | 21 |
| 4164 | BPGV22547 | Andean | ;/4 , ;/3/4 , 3/4, 4 | 10-30 | 27 |
| 4179 | BPGV22549 | Andean | ;/4 , 3/4 | 10-20 | 18 |
| 4182 | BPGV22550 | Mesoamerican | 4 | 20-50 | 35 |
| 4185 | BPGV22551 | Andean | 4 | 5-15 | 6 |
| 4189 | BPGV22552 | Andean | ;/4 , ;/3/4 , 3/4, 3(4), 4 | 8-70 | 25 |
| 4195 | BPGV22553 | - | ;/4, 4 , 3/4 | 10-25 | 24 |
| 4295 | BPGV34974 | Andean | 3/4, 4 | 10-45 | 20 |
| 4300 | BPGV34976 | Andean | 4 | 20-80 | 37 |
| 4306 | BPGV34977 | Andean | 4 | 20-25 | 25 |
| 5249 | BPGV34978 | Mesoamerican | 4, ;/4 , 3/4, 4(3) | <1-45 | 15 |
| 5285 | BPGV22567 | Andean | 3, 4, 3(4) | 10-45 | 23 |
| 5286 | BPGV22568 | Mesoamerican | ;/4 , ;/3/4, 3/4, 4 | <1-45 | 12 |
| 5287 | BPGV22569 | Mesoamerican | 4 | <1-15 | 1 |
| 5288 | BPGV34979 | Andean | ;/3/4, 3/4, 4, 4(3), ;/4, 3 | 10-60 | 30 |
| 5289 | BPGV34980 | - | ;/4, 4, 3/4 | 25-80 | 45 |
| 5291 | Not yet attributed | Andean | ;/4 , 4 | 20-30 | 26 |
| 5292 | Not yet attributed | Mesoamerican | 3, 3(4), 3/4, 4 | 10-45 | 23 |
| 5295 | Not yet attributed | Andean | 4 | <1 - 35 | 7 |
| 5296 | Not yet attributed | Mixed | ; , ;/1 , ;/2, ;(1) | < 1 | 0 |
| 5297 | Not yet attributed | Mixed | 0, 4 | 0-20 | 3 |
| 5298 | Not yet attributed | Andean | 3/4, 4 | 25-80 | 47 |

| | | | | | |
|---|---|---|---|---|---|
| 5300 | BPGV22571 | Andean | 4 | 1-10 | 2 |
| 5302 | BPGV22572 | Mixed | ;/4 , 4 | 10-25 | 23 |
| 5306 | Not yet attributed | - | 4(;), 4, 3/4 | 5-60 | 23 |
| 5363 | BPGV34982 | Andean | 3, 4, 3/4 | 15-50 | 29 |
| 5366 | BPGV22578 | Andean | ;/4 , 4 | 5-20 | 17 |
| 5367 | BPGV34983 | Andean | ;/4, 4, 4(3) | 30-70 | 44 |
| 5368 | BPGV34984 | Mixed | ;/4, 3/4, 4 | 5-60 | 15 |
| 5369 | BPGV34985 | Andean | 4 | 5-35 | 12 |
| 5370 | BPGV22579 | Mesoamerican | 4 | 5-55 | 38 |
| 5371 | BPGV34986 | Andean | 3, 3/4, 4 | 15-20 | 14 |
| 5372 | BPGV34987 | Andean | 4 | 1-10 | 2 |
| 5376 | BPGV22580 | Mixed | ;/1/2/4, ;/1/4, ;/1(4), ;/4 | <1-10 | 1 |
| 5377 | BPGV22581 | Andean | 3/4 , 4(3), 4, ;/4, ;(4) | 2-60 | 17 |
| 5378 | BPGV34990 | Mixed | 3/4, ;/4, 4 | <1-40 | 16 |
| 5379 | BPGV34991 | Mixed | ;/4 , 3/4 , 4 | 10-30 | 22 |
| 5381 | BPGV34993 | Andean | ;/2/4 , ;/4 , 4 , ;(4) | 5-15 | 12 |
| 5383 | BPGV34995 | Andean | ;/4 , 4 | 1-15 | 11 |
| 5384 | Not yet attributed | Andean | 3/4 , 4 | 10-25 | 20 |
| 5385 | Not yet attributed | Andean | ;/4 , ;(4), ;/1/4 , ;/1 , ;/1(4) | 1-15 | 9 |
| 5386 | Not yet attributed | Andean | ; / 4 | 10-30 | 26 |
| 5387 | Not yet attributed | Mixed | 3/4 , 4 , 4(3) | 15-30 | 23 |
| 5388 | Not yet attributed | Mixed | ;/4 , 4 | 5-20 | 16 |
| 5389 | Not yet attributed | Andean | 2/4 , ;/4 , ;/2/4 , 3/4 , 4 | 2-30 | 16 |
| 5391 | Not yet attributed | Andean | ;(4) , ;/1 , ;/1(4) | 1-10 | 6 |
| Tarrestre | Not yet attributed | Mesoamerican | 3/4 , 4 | 2-50 | 12 |
| SER16 | CIAT seedbank | Mesoamerican | 0, 2 | <1 | 2 |
| Tio Canela-75 | CIAT seedbank | Mesoamerican | 0, 2 | <1-5 | 3 |

[1]The common bean collection PRT005 held at the campus of INIAV in Oeiras was recently moved to the Portuguese Plant Germplasm Bank (BPGV, INIAV, Braga, website: https://www.iniav.pt/bpgv) and the process of attributing a deposition accession number in the national Bank is currently ongoing.
[2]From Leitão ST *et al* (2017) https://doi.org/10.3389/fpls.2017.01296
[3] More than one infection type in each accession, separated by commas, means that plants from the same accession presented different infection types. The slash (/) between infection types means that the same plant had mixed reactions. The

parenthesis () surrounding an infection type means an infection type present in lower abundance. <1 stands for vestigial sporulation and trace disease symptoms.

A

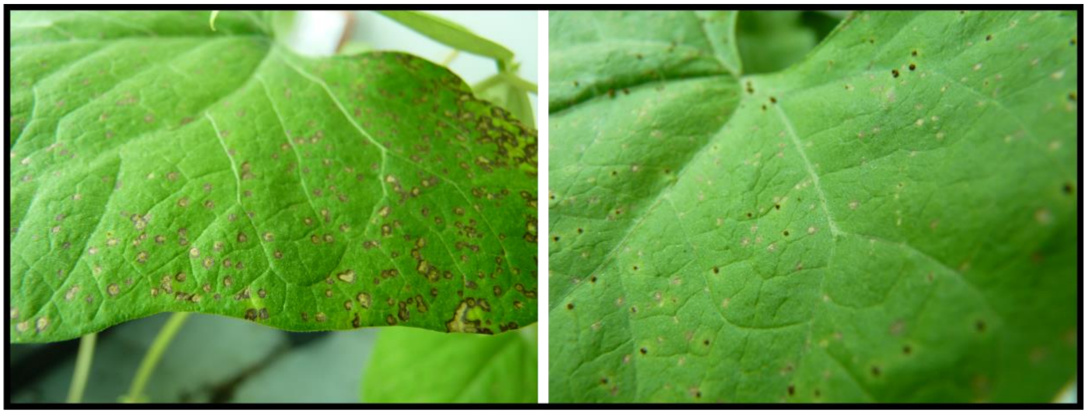

Incomplete hypersensitive-resistance
(low IT and low DS)

B

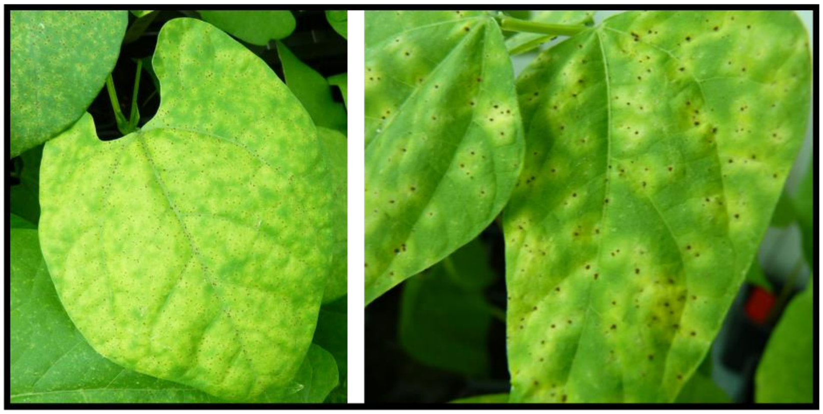

Partial resistance (high IT and low DS)

**Figure S1 -** Examples of incomplete hypersensitive resistance (A) and partial resistance (B)

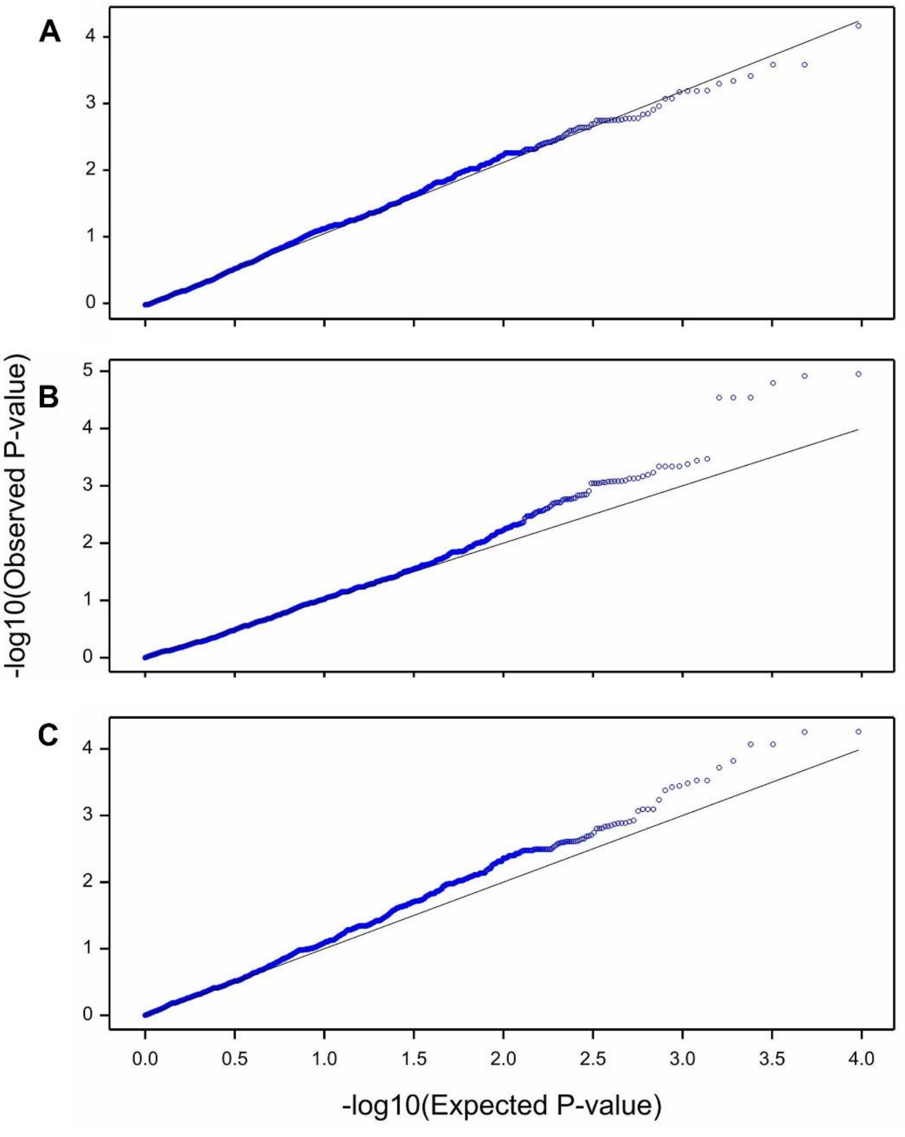


**Figure S2 -** Quantile-quantile (QQ) plots corresponding to the association mapping analyses using a linear mixed model with one kinship matrix per chromosome to account for the genetic relatedness among accessions. A – association panel with all the 132 common bean accessions. B – association panel after removing the 6 accessions considered incomplete hypersensitive resistant, to detect genes responsible for partial resistance. C – association panel after removing the 20 partially resistant accessions, to detect genes responsible for incomplete hypersensitive resistance.
